# Optimization of Irreversible Electroporation Protocols for In-vivo Myocardial Decellularization

**DOI:** 10.1371/journal.pone.0165475

**Published:** 2016-11-28

**Authors:** Yaniv Zager, David Kain, Natalie Landa, Jonathan Leor, Elad Maor

**Affiliations:** 1 Tamman Cardiovascular Research Institute, Sheba Medical Center, Tel-Hashomer, Ramat-Gan, Israel; 2 Sackler School of Medicine, Tel-Aviv University, Tel-Aviv, Israel; 3 Pinchas Borenstein Talpiot Medical Leadership Program, Sheba Medical Center, Tel-Hashomer, Ramat-Gan, Israel; Consiglio Nazionale delle Ricerche, ITALY

## Abstract

**Background:**

Irreversible electroporation (IRE) is a non-thermal cell ablation approach that induces selective damage to cell membranes only. The purpose of the current study was to evaluate and optimize its use for in-vivo myocardial decellularization.

**Methods:**

Forty-two Sprague-Dawley rats were used to compare myocardial damage of seven different IRE protocols with anterior myocardial infarction damage. An in-vivo open thoracotomy model was used, with two-needle electrodes in the anterior ventricular wall. IRE protocols included different combinations of pulse lengths (70 vs. 100 μseconds), frequency (1, 2, 4 Hz), and number (10 vs. 20 pulses), as well as voltage intensity (50, 250 and 500 Volts). All animals underwent baseline echocardiographic evaluation. Degree of myocardial ablation was determined using repeated echocardiography measurements (days 7 and 28) as well as histologic and morphometric analysis at 28 days.

**Results:**

All animals survived 28 days of follow-up. Compared with 50V and 250V, electroporation with 500V was associated with significantly increased myocardial scar and reduction in ejection fraction (67.4%±4% at baseline vs. 34.6%±20% at 28 days; p <0.01). Also, compared with pulse duration of 70 μsec, pulses of 100 μsec were associated with markedly reduced left ventricular function and markedly increased relative scar area ratio (28%±9% vs. 16%±3%, p = 0.02). Decreasing electroporation pulse frequency (1Hz vs. 2Hz, 2Hz vs. 4Hz) was associated with a significant increase in myocardial damage. Electroporation protocols with a greater number of pulses (20 vs. 10) correlated with more profound tissue damage (p<0.05). When compared with myocardial infarction damage, electroporation demonstrated a considerable likeness regarding the extent of the inflammatory process, but with relatively higher levels of extra-cellular preservation.

**Conclusions:**

IRE has a graded effect on the myocardium. The extent of ablation can be controlled by changing pulse length, frequency and number, as well as by changing electric field intensity.

## Introduction

Electroporation is a biophysical phenomenon in which cell membrane permeability to ions and molecules increases significantly in response to externally applied short pulses of direct current electric fields. When the electric field applied is of relatively strong intensity, it may induce persistent change in membrane permeability and lead to cellular death. This process is called irreversible electroporation (IRE) and is an emergent non-thermal cell ablation modality that is increasingly used clinically to treat solid tumors [1–3]. Recent studies (including human trials) suggest that IRE can be used as adjuvant treatment for various solid malignancies such as: prostate [4], pancreas [5] [6] and liver [7]. Due to its simplicity of use, its non-thermal nature, and its ability not to damage extra-cellular components such as blood vessels [8–10], it is being evaluated in pre-clinical studies as a new approach for cardiovascular tissue ablation [11–13].

IRE holds the potential to become an important transcatheter modality in both invasive electrophysiology and interventional cardiology. In the field of cardiac arrhythmias, the unique ability of IRE to induce an extensive and precise ablation zone with no thermal damage offers important advantages for both pulmonary veins and ventricular ablations, since its non-thermal nature can be translated into reduced risk of complications, such as pulmonary vein stenosis, coronary damage and tissue perforation [12–15]. In the field of structural heart disease, IRE holds the potential to attenuate outflow tract obstruction in hypertrophic cardiomyopathy by ablating septal myocardium using a minimally invasive approach.

Electroporation protocols include many parameters that can be controlled. These parameters include pulse length, electric field intensity and distribution, the number of pulses applied and their frequency. Each of these parameters can have a significant influence on the efficiency of electroporation treatment. In addition, the effect of electroporation is also highly dependent on electric field distribution and its orientation with respect to cell membranes [15]. Therefore, electroporation protocols must be tailored according to the target organ and tissue, and results of pre-clinical studies of one tissue or organ should not be used to plan and treat other tissues.

The hypothesis of the current study was that IRE is a safe and efficient modality for in vivo myocardial decellularization, and that it has a graded effect that can be controlled by modifying electroporation protocols. Study objectives are therefore: (1) to evaluate the safety of in vivo IRE in a rodent model (2) to evaluate and compare the potency and graded effect of different electroporation protocols on in vivo myocardial tissue.

## Methods

### Electroporation protocols

**Fig 1:**
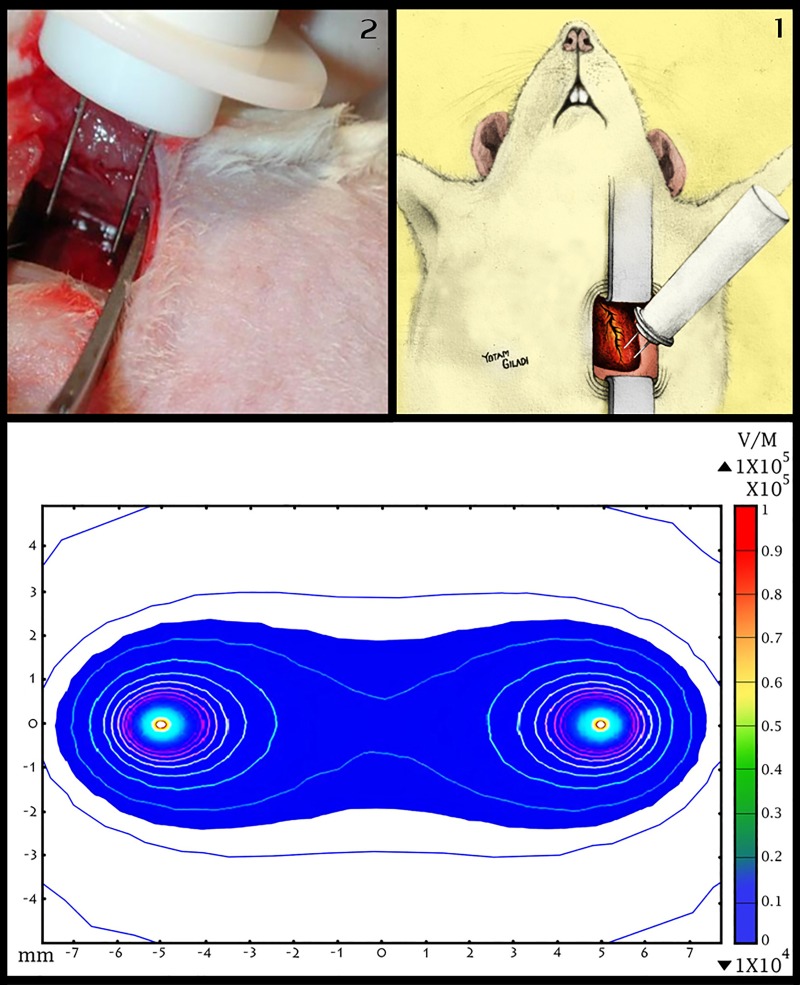
Animal model and electric field distribution After sterile thoracotomy, IRE was applied to the anterior myocardium located to the left of the lower part of the left anterior descending artery (Fig 1.1 and Fig 1.2). Electric field distribution induced by two-needle electrodes and a 500 volt pulse are illustrated in Fig 1.3. Note that electric field intensity is concentrated in the plane between the two electrodes.

A BTX ECM 830 (Harvard Apparatus, Holliston, MA, USA) electroporation pulse generator was used in this experiment. The generator was connected to two-needle electrodes (distance of 10mm between electrodes, the actual diameter of electrodes is unknown exactly according to the manufacturer, however it is significantly small in comparison with the distance between the electrodes and therefore can be neglected), and the electric field was concentrated in the volume around and between the two electrodes (Fig 1). The pulse generator was used to control and modify four electroporation parameters: pulse duration, pulse number, pulse frequency and pulse voltage intensity. Seven different protocols of electroporation were used. Protocols 1–3 used similar pulse number, frequency and duration, but were different with respect to the voltage intensity used (50V, 250V and 500V). In addition, in order to allow for a better understanding of electroporation protocol settings, four additional protocols (5–8) were used with 500V but with variations in pulse duration, pulse number and pulse frequency. A complete list of the electroporation protocols used in this study is shown in Table 1. All protocols were simulated and evaluated using COSMOL MULTIPHYSICS 4.2a in order to validate that no significant thermal damage was induced [16] The effects of the seven electroporation protocols were compared between the different protocols, using an anterior myocardial infarction (MI) model as a positive control (see below). Anterior MI was used as a positive control due to the extensive anterior wall scar it induces, and also because of the extensive literature and experience with this animal model.

**Table 1:** Electroporation protocols used in the study.

Protocol #	Pulses	Frequency	Pulse duration	Voltage
1	10	1 Hz	100μsecs	50 V
2	10	1 Hz	100μsecs	250 V
3	10	1 Hz	100μsecs	500 V
4	10	2 Hz	70μsecs	500 V
5	10	1 Hz	70μsecs	500 V
6	20	2 Hz	70μsecs	500 V
7	20	4 Hz	70μsecs	500 V
8	Anterior myocardial infarction			

While all IRE protocols use the same needle-electrode configuration, they differ in at least one of four properties: number of pulses, pulse frequency, pulse duration and voltage difference. In addition to seven IRE protocols, an anterior MI group was included in the study as a positive control.

### Animal Model and Intervention

Female Sprague Dawley rats (N = 45, 270±21 grams) were obtained from Harlan Laboratories Ltd., Jerusalem Israel. Rats were housed in regular cages situated in an animal room at a constant temperature of 22°C with a 14-h light/10-h dark cycle. Rats were maintained on a standard rat chow diet and given tap water to drink for at least five days prior to the intervention in the lab. All experiments were approved by the ethical committee of the Chaim Sheba Medical Center (approval numbers 751/12/ANIM and 767/12/ANIM). All animals underwent baseline echocardiographic evaluation at day 0, before any intervention was performed. Intramuscular injection of ketamin (35mg/kg body weight) and xylazine (5 mg/kg body weight) was used for anesthesia, followed by endotracheal intubation and mechanical ventilation. We used a sterile left thoracotomy approach at the fourth intercostal space level which, after palpation, was estimated to be next to the apex. The "surgical window" between the ribs was stabilized by an appropriate retractor. Using a sterile approach, two-needle electrodes were introduced into the anterior wall of the myocardium via the intercostal window. The electroporation needles were introduced into the anterior wall after careful identification of the left anterior descending (LAD) artery near the apex. The electrodes were placed 3mm left of the LAD and 2mm above the left ventricular apex. The needle electrodes were inserted deep into the myocardial tissue in order to ensure maximal effect of the electric field (Fig 1.2 and Fig 1.3). Electroporation pulses were then applied with the use of a high voltage pulse generator as described above. Following pulse delivery, air was drained from rats' chests by manual maneuver and the skin was sutured. Rats were then given fluids and dipyrone subcutaneously. During the follow-up period animals were kept in the Sheba Medical Center animal facility under veterinarian supervision and were screened daily. It should be noted that apart from surgical knots on the rats' skin, no other adverse effects were observed during follow-up. Staff from the study's team treated these surgical knots by suturing the skin, while rats were again subjected to anesthesia.

The MI group animals (protocol 8) underwent LAD ligation in order to induce infarction of the anterior wall using the same surgical approach. We identified the LAD 2-3mm after its branching out from the left main coronary artery. It was ligated by the introduction of a 0–6 nylon surgical knot into the tissue, thereby creating a double knot with pressure on the LAD. Successful occlusion of the LAD was verified by a change in color of the infarcted area immediately following the ligation. At day 28, all animals underwent final echocardiography and were then euthanized with an injection of high-dose potassium chloride after being sedated by a ketamin and xylazine intamuscluar injection, and inhalation of isoflurane.

### Echocardiographic measurements

Echocardiographic studies were performed at baseline (day 0) and on days 7 and 28 (immediately prior to euthanasia) by a blinded operator. Two-dimensional transthoracic echocardiographic and Doppler studies were obtained with VisualSonicsVevo 2100® system (Fuji-Film Visual Sonic Inc., Toronto, Ontario, Canada). All studies were digitally stored and included evaluation of left ventricular ejection fraction (EF) and fractional shortening (FS). Both measures of left ventricular systolic function were used to evaluate systolic function in each animal at each time point, in order to enhance the consistency and accuracy of left ventricular function.

### Histology and morphometric measurements

Following euthanasia hearts were perfused with 4% formaldehyde solution, harvested and cut into three slices: base, mid and apical segments. This was followed by preparation of histological slides that were stained for hematoxylin-eosin (H&E), and digitally photographed with a scale adjacent to the slide. The final images were stored digitally for further morphometric analysis. Histology and morphometric analysis were used to evaluate the extent of inflammation, fibrosis and volume reduction. For each animal we chose one slide out of 3 (basal, mid or apical) that was optimal in means of demonstrating maximal tissue damage and a clear demarcation line between scar and healthy tissue. Morphometric measurements were done for all histologic slides, and included the area, perimeter and thickness of scarred and normal myocardium. Later, using SigmaScan Pro version 5 software (Image Analysis ®), a ratio between the scar and healthy part of the left ventricle was calculated for thickness (by calculating the ratio between the average of three measurements of scar thickness and 3 measurements of healthy tissue thickness; for the perimeter (by marking a line through the scar [which was located approximately between the epicardium and the endocardium], and then measuring its length using the software, after that a similar line was drawn through the entire left ventricle. Following this, its length was measured using the software, and then the ratio between the lengths of the two lines was calculated); and for the area (by marking the contour of the damaged portion of the tissue and then calculating the scar's area using the software. After this, the area of the entire left ventricle was measured in the specific histological preparation in the same way, and then the ratio between the damaged area and the entire left ventricular area was calculated). Masson Trichrome stain was used in borderline cases where the border between normal and damaged tissue could not be evaluated using the standard H&E stain. ED1 and collagen staining were used to evaluate qualitatively the extent of inflammation and fibrosis.

### Statistical Analysis

Echocardiographic parameters are presented as average and standard deviation and were calculated for days 0, 7 and 28. Morphometric measurements are presented as average and standard deviation. Initially, echocardiographic data were compared using two way repeated measures ANOVA, Turkey's correction was used to asses significance of predefined comparisons in specific points of time. Later, echocardiographic and morphometric data of pairs of protocols were compared by unpaired Student t-test and echocardiographic data of protocols in different time points of the study were compared by paired t-test. The statistical analyses were performed with Graphpad Prism version 7.0 (California, USA). Statistical significance was defined as p<0.05.

## Results

**Fig 2:**
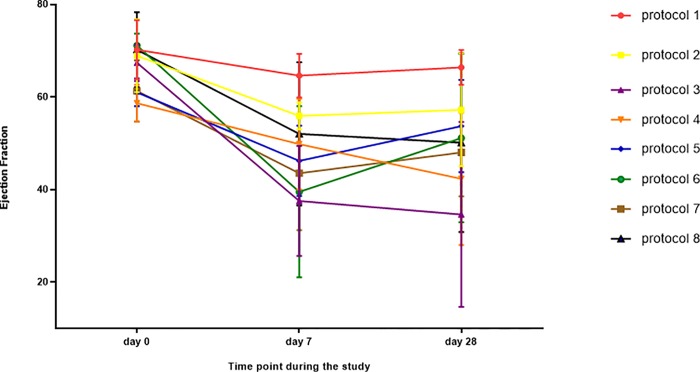
Average ejection fraction of various study groups during the study period. The vertical axis stands for ejection fraction (EF) in %. The horizontal axis stands for time point during the study. Each group was treated with a different IRE protocol. Note that the reduction in EF was demonstrated in all the protocols. Some of the groups demonstrated some recovery in EF between day 7 and day 28. Some of the IRE protocols caused more severe damage than the anterior MI group (protocol 8).

**Fig 3:**
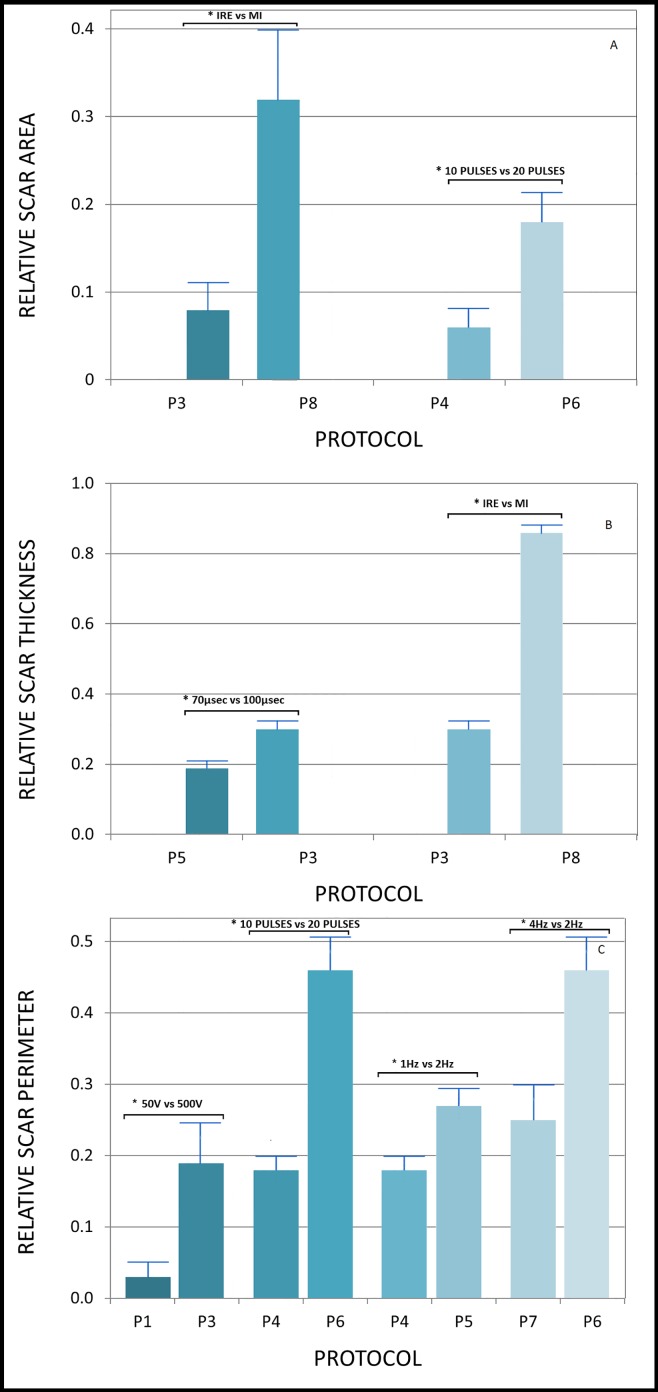
Comparison of morphometric measurements between pairs of protocols. Morphometric measurements of pairs of protocols with only one changed parameter were compared (using unpaired Student t-test), in order to learn about the effect of different parameters on the potency of each protocol. Only pairs with a statistically significant difference in morphometric measurements are shown. From top to bottom: (3a) presents comparisons of scar areas with significant differences. (3b) presents comparisons of scar thicknesses with significant differences. (3c) presents comparisons of scar perimeters with significant differences.

**Fig 4:**
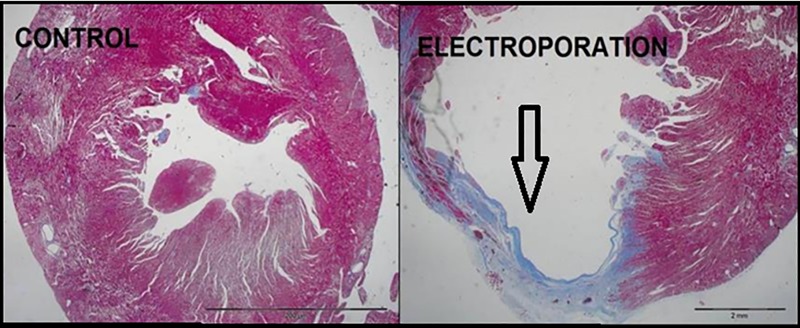
Microscopic view of collagen staining of rat hearts. The collagen staining demonstrates the presence of extended scar tissue in the myocardium that has been treated by IRE. The control slide (left figure) lacks bluish coloring because of lack of scar. In the right figure, the bluish coloring demonstrates well the typical scaring caused by IRE as was observed in our study.

**Fig 5:**
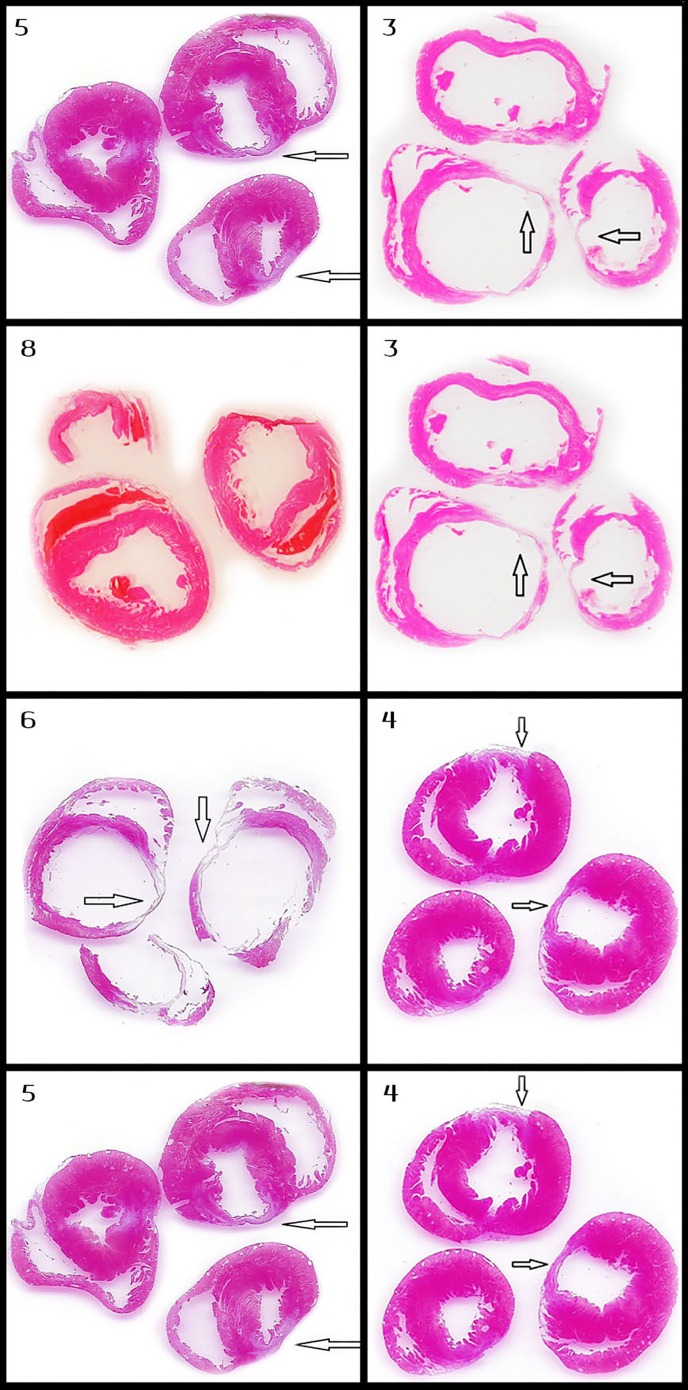
Comparison of myocardial axial section of different protocols. Every row in the figure compares the axial section of the left ventricle of rats that were treated with two different IRE protocols. Moreover, all the pairs of protocols selected for this figure are different in only one setting of the IRE protocols (i.e. voltage, frequency, etc.) and demonstrate significant differences between their morphometric measurements. Protocol numbers are in the right upper corner of each slide. The pairs of protocols are (from top to bottom): 5 and 3(70μsec and 100μsec), 8 and 3 (MI vs. IRE), 6 and 4 (10 pulses vs. 20 pulses), 5 and 4 (2Hz vs. 1HZ).

**Fig 6:**
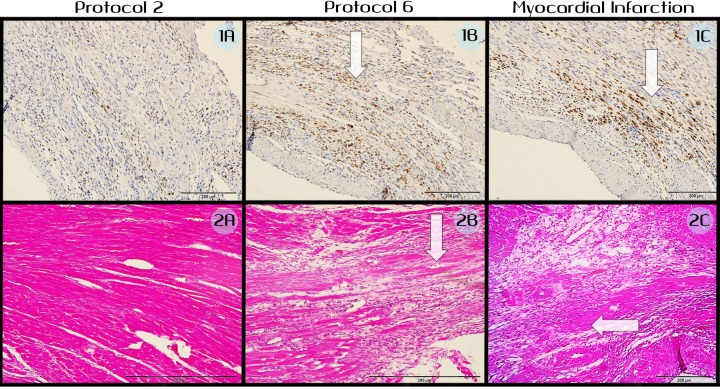
Microscopic view of treated myocardium. In each row the images from right to left represent: low potency IRE protocol (protocol 2), high potency electroporation protocol (protocol 6), MI group (protocol 8). ED1 staining is presented in the upper row, while H&E staining is presented in the lower row. Note: 1) the similar extent of damage between high potency IRE protocol and MI–images 1B and 1C, 2B and 2C. 2). The greater degree of extracellular damage caused by MI compared with low and high potency IRE protocols–images 2C, 2A, 2B respectively. Arrows point to the areas of extensive inflammation.

The final study included 45 rats. Three animals died during the surgical and pre-procedural period: one during induction of anesthesia, one during traumatic intubation and one as a result of laceration of LAD during resection of the pericardium. There was no mortality during follow-up, and all 42 animals survived the procedure and 28 days of follow up. It should be noted that in the lack of ECG recording, in some of the rats, alternation in rhythm (into irregular) has been observed with the naked eye. However, in echocardiographic evaluation in the day 7 all hearts seemed to have regular rhythm. Echocardiographic parameters for days 0, 7 and 28 presented in Table 2 and Fig 2. Notably, most echocardiographic studies showed a trend of decrease in LV function both in day 7 and day 28. Ejection fraction was: 65.83%±6.18%, 48.08%±14.08% and 50.17%±16.77% in days 0, 7 and 28 respectively (p<0.001). Analysis of echocardiographic damage has demonstrated significant difference between protocols during study's time points in means of EF (P = 0.02) as well as FS (P<0.01). Protocol 3 has demonstrated the most profound tissue damage both in both echocardiographic and morphometric aspects and also protocol 6 seem to be highly potent. In contrast, protocols 2, 4 and 7 seems to have relatively low potency and protocol 1 seems to have the lowest potency. Morphometric measurement data are summarized in Fig 3 and selected specimens are presented in Fig 4, Fig 5 and Fig 6.

**Table 2:** Summary of echocardiographic measurements.

	Protocol1 (N = 5)	Protocol 2 (N = 3)	Protocol 3 (N = 5)	Protocol 4 (N = 5)	Protocol 5 (N = 5)	Protocol 6 (N = 6)	Protocol 7 (N = 4)	Protocol 8 (N = 6)
Ejection fraction (%)								
• Baseline	70.2±6.4	68.9±7.9	67.4±4	58.65±4	61±3	71.1±2.6	61.3±6.6	70.2±8.1
• Day 7	64.6±4.7	55.9±2.9	37.5±11.9	49.8±9.8	46.2±7.6	39.5±18.5	43.5±12.3	52±15.5
• Day 28	66.4±3.8	57.2±15.2	34.6±20	42.3±14.31	53.7±10	51.1±18.2	48±9.8	50.1±19.3
Change (%) *	5.4	17.0	49.7	27.9	12.0	28.1	21.7	28.6
P value **	0.25	0.53	<0.01	0.78	0.03	0.01	0.07	0.04
Fractional shortening								
• Baseline	41.3±5.6	37.2±6.3	34.82±8.5	32.08±2.5	33.7±2.2	41.5±2.2	34.1±4.8	41.6±7.6
• Day 7	36.8±4.7	30.4±2	19.21±6.7	26.4±6.4	24±4.6	20.9±11	22.8±7.3	28.4±10
• Day 28	38.2±3	32±11.1	18.3±12.1	22.1±8	29.2±6.8	28.1±11.6	25.4±6.1	27.8±12.8
Change (%) *	7.5	14.0	47.4	31.1	13.4	32.3	25.5	33.2
P value **	0.27	0.54	0.01	0.68	0.03	0.01	0.07	0.04

For each of the study groups, ejection fraction (EF) and fractional shortening (FS) were evaluated at time points during the study: at baseline (prior to intervention), 1 week post-intervention and at the end of follow-up, 28 days after intervention. Change is between baseline and day 28.

** Paired t-test was used to compare baseline and day 28 measurements.

Significant P-values (P < .05) were marked in asterisk.

### Graded effect of voltage

Protocols 1, 2 and 3 differed only in voltage of pulses: 50, 250, and 500. Notably, only protocol 3 demonstrated significant echocardiographic changes (EF change was 48.66%, p<0.01. FS was 47.44%, p = 0.01, Table 2). In addition, direct comparison of EF changes between protocols 1 and 3 demonstrated a significant difference (p = 0.03, Table 3). Also, morphometric analysis supported this trend as demonstrated by direct comparison of the scar's relative perimeter between protocol 1 and 3 (0.19±0.05 vs. 0.02±0.02, p = 0.01, Fig 3).

**Table 3:** Comparison of changes in ejection fraction between protocols.

Protocols compared	1 vs. 2	1 vs. 3	2 vs. 3	3 vs. 5	3 vs. 8	4 vs. 5	6 vs. 7	4 vs. 6
Parameter type changed	Voltage	Voltage	Voltage	Pulse length	Ischemia	Pulse frequency	Pulse frequency	Pulse number
Values compared	50 vs. 250V	50 vs. 500V	250 vs. 500V	70 vs. 100 μsec	Protocol 3 vs. MI	1 Vs. 2 Hz.	2 vs. 4 Hz.	20 vs. 10 pulses
P value of EF comparison	0.3	0.02*	0.13	0.06	0.35	0.23	0.75	0.84
P value of FS comparison	0.35	0.09	0.36	0.21	0.03*	0.21	0.75	0.89

The changes in EF and FS on day 28 following intervention were compared between pairs of protocols and were different in only one parameter in order to isolate the effect of changing certain parameters on functional damage. Note that only voltage had a significant effect on EF (protocol 1 vs. 3). Longer pulse length demonstrated an intermediate effect on EF (protocol 3 vs. 5). FS changed significantly only between IRE by protocol 3 compared with MI. Significant P-values (P < .05) were marked in asterisk.

### Graded effect of pulse length

Protocols 3 and 5 differed in pulse length only: from 100 μsec in protocol 3, to 70 μsec in protocol 5. Notably, EF was reduced by only 12% (from 61%±3% to 53.7%±10%; p = 0.03, Table 2) in protocol 5, compared with almost 50% in protocol 3. FS study also demonstrated higher potency of protocol 3 (from 34.82%±8.5% to 18.3%±12.1%; p = 0.01, Table 2) compared with protocol 5 (from 33.7%±2.2% to 29.2%±6.8%; p = 0.03, Table 2). Also, morphometric analysis demonstrated more extensive scar caused by protocol 3 regarding thickness ratio (0.28±0.03 vs. 0.16±0.01, p = 0.04, Fig 3).

### Effect of pulse frequency

In order to investigate tissue damage dependence on electroporation frequency/treatment duration, we tested two sets of protocols that were identical with the exception of pulse frequency. Protocol 5 (1Hz) was compared with protocol 4 (2Hz), and protocol 6 (2Hz) with protocol 7 (4Hz). Lower frequency protocols (5 and 6) demonstrated significant echocardiographic evidence of tissue damage regarding both EF and FS reduction, while the higher frequency protocols (4 and 7) did not demonstrate any significant reduction in echocardiographic measures (Table 2). For example, protocol 5 group demonstrated significant EF reduction from 61%±3% to 53.7%±10% (p = 0.03), while protocol 4 did not demonstrate significant change in EF during the study period. This trend was also supported by morphometric measurements which demonstrated a larger relative scar perimeter of protocol 5 compared with protocol 4 (0.28±0.02 vs. 0.18±0.02, p = 0.01, Fig 3) and a larger relative scar perimeter of protocol 6 compared with protocol 7 (0.46±0.07, 0.25±0.05, p = 0.03, Fig 3).

### Graded effect of pulse number

In order to investigate the dependence between tissue damage and the number of pulses, we used two protocols with similar electroporation properties, except for the number of pulses: 10 pulses in protocol 4 and 20 pulses in protocol 6. While at 28 days, protocol 4 showed non-significant EF and FS reduction, protocol 6 correlated with significant EF reduction (from 71.1±2.6 to 51%±18.2%, p = 0.01, Table 1) and significant FS reduction (41.5±2.2 vs. 28.1±11.6, p = 0.01). In terms of scar morphology, protocol 6 caused more extensive damage with regard to relative scar area (protocol 6: 0.18±0.05, protocol 4: 0.06±0.02, p = 0.03, Fig 3) and relative scar perimeter (0.46±0.07 vs. 0.18±0.02, p<0.01, Fig 3).

### IRE versus MI

In order to study the differences between ischemic and Electroporation -induced tissue damage, we compared the MI group with the electroporation protocol that was found to be the most potent in the study–protocol 3. Protocol 3 reduced EF by 44% (p<0.01), while EF in the MI group was reduced by 28% (p = 0.04; Table 2). However, FS measurements demonstrated a reduction of 33.17% in protocol 3 (p = 0.04) vs. 47.44% in the MI group (p = 0.01). Also a direct comparison between FS changes in protocol 3 vs. the MI group was significant (p = 0.03, Table 3). The morphometric measurements demonstrated a significant smaller scar in protocol 3 compared with the MI group regarding relative scar thickness and area. The relative scar area in protocol 3 was 0.08±0.03, while in MI it was 0.32±0.0.08; p = 0.02, Fig 3). Regarding the comparison of qualitative properties of the inflammatory process and scaring: Fig 6.1 demonstrates a considerable likeness regarding the extent of the inflammatory process between the MI and high potency electroporation protocol specimens.

## Discussion

The main finding of the current study is that electroporation-induced myocardial damage can have a graded effect with the same electrode configuration. The extent of damage can be controlled by changing pulse number, frequency, duration or voltage. To the best of our knowledge, this is the largest small animal study that has systematically evaluated IRE protocols in an in vivo beating heart model with long-term follow-up.

Consistent with previous studies of other tissue organs, our study has again demonstrated the safety and efficiency of IRE as a non-thermal non-pharmacological cell ablation approach. Despite our aggressive protocol, that included high voltage output through two needles that were in direct contact with the anterior myocardium, all animals that underwent IRE survived 28 days of follow up. Echocardiography measurements have demonstrated a more extensive damage on day 7 in comparison with day 28. We believe that this finding reflects the temporal recovery and remodeling of the left ventricular myocardium following IRE insult.

By comparing and examining seven different IRE protocols, our study demonstrates four important issues: 1) Longer pulse duration (100 μs vs. 70 μs) is associated with larger volume reduction; 2) More pulses (20 vs. 10) are associated with larger volume reduction; 3) Pulse voltage (500V vs. 250V, 50V) has an important effect on tissue damage; 4) Lower pulse frequency (10Hz vs 20Hz) is correlated with harsher tissue damage, most likely due to the longer overall treatment duration time. From the clinical perspective, the use of high output, longer pulse duration and a larger number of pulses can be used to increase tissue damage with the same electrode configuration (and vice versa). Also, when comparing the ischemic damage to the damage caused by the highest potency electroporation protocol, our results show that electroporation was associated with more extensive damage.

While electroporation studies are difficult to compare due to high variability in the description of electric field used, our findings are consistent with previous studies evaluating the effect of IRE pulses on cardiovascular structures. In study of large blood vessels in a rodent model with two-plate electrodes, ninety 100-μsec electroporation pulses of 1,750 V/cm at a frequency of 1 Hz were associated with complete decellularization of the arterial wall [17]. Our study, which used similar electroporation pulses extends the results of that study and shows their validity in a beating heart model.

Lavee et al. were the first to demonstrate the potential of IRE as an epicardial ablation modality. In their short-term (24 hours) study of 5 pigs, atrial tissue was successfully ablated with IRE pulses of 1,500–2,000 volts using parallel needle electrodes. Their work successfully showed transmural tissue damage without peripheral thermal damage. [11]

Neven and colleagues successfully studied the safety of IRE epicardial use by purposely targeting epicardial coronary arteries in a beating heart pig model (N = 5). In their study, 200J were applied using a circular catheter with 2-mm ring electrodes. Coronary angiography at 3 months demonstrated similar luminal diameter, with the important clinical conclusion that IRE can create deep lesions and is a safe modality for catheter ablation on or near coronary arteries. [12] In a separate study with a similar electrode design, Neven and colleagues showed the graded effect of electroporation pulses by using 50, 100, and 200 J electroporation pulses in 6 pigs that were followed up for 3 months. In their study, increasing energy was associated with increase in both lesion depth and thickness. [13]While most electroporation protocols use pulse duration around 100μsec, Xie and colleagues successfully demonstrated the efficiency of nanosecond pulses in an ex-vivo Langendorff-perfused New Zealand rabbit hearts (n = 12). [18]

While all IRE studies of in-vivo hearts were done epicardially, IRE can also be successfully used in a trans-catheter, endovascular approach. In a small study of New Zealand white rabbits (N = 8), electroporation protocols that were very similar to those used in our current study were applied using an endovascular approach and induced efficient ablation of the iliac arterial wall. [19] We hypothesize that in addition to possible role in arrhythmias treatment this transcatheter endovascular approach of IRE might also prove to be useful in the future in hypertrophic cardiomyopathy patients of at high surgical risk who are in need of myectomy or any other surgical procedure that involves removal of ventricular tissue.

### Limitations

First, electrocardiograms were not documented during the procedures or during the follow-up period. Therefore, we cannot rule out that IRE treatments were associated with malignant ventricular arrhythmias. Moreover, we believe that further evaluation of IRE based cardiac-treatment should include observation on intra-procedural cardiac functioning. Second, since two- needle electrode configuration was used in this study, our results might not be valid in other electrodes design. There were also some limitations regarding measurements and data-analysis: 1) the lack of assessment of extracellular damage and preservation and 2) the fact that due to lower quality or a small scar, not all the histological slides of each animal in the morphometric evaluation. Finally, since electroporation effect is dependent on cell shape and orientation, our results cannot be extended to human hearts with common pathological conditions, such as ischemic cardiomyopathy, dilated cardiomyopathy or hypertrophic cardiomyopathy. Further studies are therefore warranted before IRE can be considered for use in the treatment of these clinical conditions in human, and direct comparison of IRE to commonly used myocardial ablation modalities such as Radiofrequency ablation is warranted.

## Supporting Information

S1 Dataset Echocardiographic data. Each column describes one echocardiographic measurement. The name of the measurement is indicated in the first line and includes a number which is the serial number of the rat and a letter which represents the timing of measurement (A = day 0, B = day 7, C = day 28). The second line contains the number of the IRE protocol that has been applied on the rat during the experiment. The third line of each column is the Ejection Fraction presented in percent. The fourth line is the Fractional Shortening presented in percent.
(XLSX)

S2 Dataset Morphometric data. Each line represents certain rat and contains morphometric data and analysis based on histological preparations made in the end point of the study (the 28^th^ day). The first column contains the serial number of the rat. The second column contains the IRE protocol number that has been applied on the specific rat. The next column contains comments made during the measurements regarding technical qualities of the preparations. The next three columns contain measurements of thickness of the scar (as explained in the Methods sections). The next column contains the average thickness of scar for each animal. In the same manner the next four columns contains the thickness of healthy myocardial Left Ventricle tissue and average thickness of healthy myocardial tissue. The next column contains the ratio between average thickness of myocardial scar tissue and average thickness of healthy myocardial tissue. The next column contains the perimeter of scar tissue in each preparation (measurement technique is described in the Methods section). The next column contains the perimeter of the whole Left Ventricle in each preparation. The next column contains the relative perimeter of scar from the Left Ventricle. In the same manner, the next three columns contains the area of scar (measurement technique is described in the Methods section), area of Left Ventricle and the relative area of scar from the Left Ventricle.
(XLSX)
